# Dynamic structures and emerging trends in the management of major trauma: A bibliometric analysis of publications between 2012 and 2021

**DOI:** 10.3389/fpubh.2022.1017817

**Published:** 2022-11-01

**Authors:** Zhe Du, Zhenzhou Wang, Fuzheng Guo, Tianbing Wang

**Affiliations:** Trauma Center, Peking University People's Hospital, National Center for Trauma Medicine, Key Laboratory of Trauma and Neural Regeneration (Ministry of Education), Beijing, China

**Keywords:** major trauma, management, bibliometric analysis, visualization, CiteSpace

## Abstract

**Objective:**

Major trauma is currently a global public health issue with a massive impact on health at both the individual and population levels. However, there are limited bibliometric analyses on the management of major trauma. Thus, in this study we aimed to identify global research trends, dynamic structures, and scientific frontiers in the management of major trauma between 2012 and 2021.

**Methods:**

We searched the Web of Science Core Collection to access articles and reviews concerning the management of major traumas and conducted a bibliometric analysis using CiteSpace.

**Results:**

Overall, 2,585 studies were screened and published by 403 institutions from 110 countries/regions. The most productive country and institution in this field of research were the USA and Monash University, respectively. Rolf Lefering was the most prolific researcher and Holcomb JB had the most co-citations. *Injury* published the highest number of articles, and the *Journal of Trauma* was the most co-cited journal. A dual-map overlay of the literature showed that the articles of most publications were confined to the areas of medicine/medical/clinical and neurology/sports/ophthalmology. Document clustering indicated severe traumatic brain injury, traumatic coagulopathy, and resuscitative endovascular balloon occlusion as the recent hot topics. The most recent burst keywords were “trauma management,” “neurocritical care,” “injury severity,” and “emergency medical services.”

**Conclusion:**

The dynamic structures and emerging trends in the management of major trauma were extensively analyzed using CiteSpace, a visualization software. Based on the analysis, the following research hotspots emerged: management of severe traumatic brain injury and massive hemorrhage, neurocritical care, injury severity, and emergency medical service. Our findings provide pertinent information for future research and contribute toward policy making in this field.

## Introduction

Major trauma is a life- or limb-threatening injury caused by a blunt force, penetrating injury, or burn injury. The Injury Severity Score (ISS) is a crucial element of the trauma system evaluation, with ISS scores ≥ 16 indicating major or severe trauma (1, 2). In the United States, the mortality rate of people with major trauma is 20%, and many survivors remain permanently disabled (3). Major trauma is currently a global public health issue (4), the main cause of death in the first four decades of life, and a major cause of potential loss of years of life (5). Efficient management of major trauma is of paramount importance in improving care quality and decreasing mortality (6). Many laboratory studies and clinical trials on the management of major trauma have been conducted over the last decades (7–10). However, there is a lack of summary and evaluation of publishing output trends; influential countries, regions, institutions, and authors; the current state of knowledge; and frontier trends in research related to the management of major trauma.

A bibliometric analysis is a quantitative analysis tool to examine the characteristics of literature, recent developments, and research hotspots (11). The bibliometric methodology has become popular and is increasingly being used in medical research (12). Contrary to systematic reviews and meta-analyses, bibliometric analyses aim to construct a citation network by summarizing publications using performance analysis and science mapping. Consequently, bibliometric analyses contribute toward bridging gaps in current knowledge and facilitating the creation of new directions (13). CiteSpace is an extensively used scientific software that identifies and visualizes the current knowledge domain, detects trending topics in the literature, and indicates future research directions (14). Though other popular tools such as Vosviewer and Biblioshiny exist, CiteSpace was one of the main tools used in several bibliometric analyses (15–17). Therefore, the aim of this study was to conduct a bibliometric analysis using CiteSpace to analyze the current state of knowledge, explore the evolutionary path of severe trauma management, and identify emerging trends in the management of major trauma.

## Materials and methods

### Data acquisition

**Figure 1:**
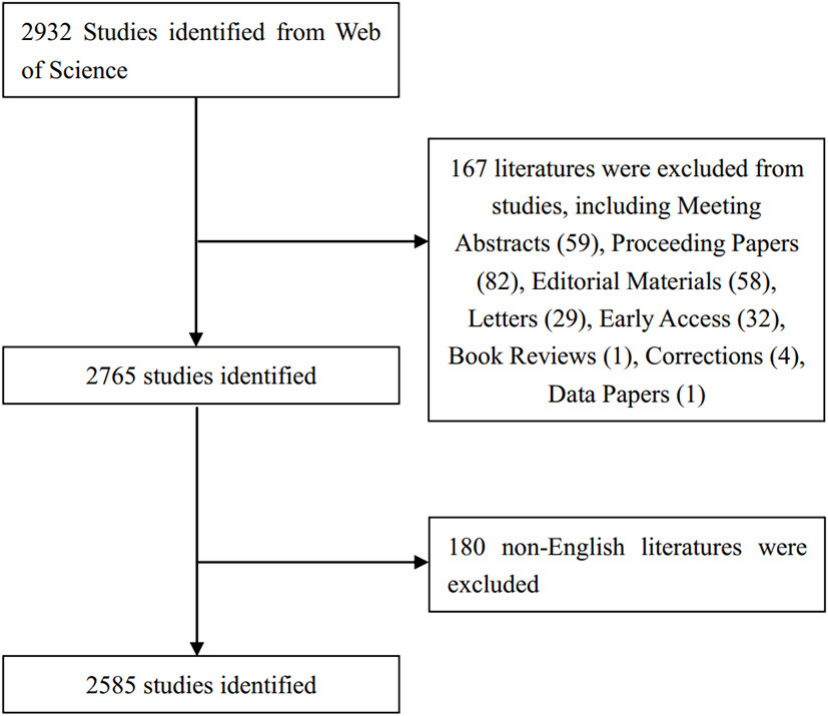
Flowchart of the screening process.

Data were retrieved from the Web of Science Core Collection (WoSCC), and the search strategy was as follows: (TI = “management”) and (TI = “major trauma^*^” OR “severe trauma^*^” OR “severe injur^*^”). The symbol “^*^” was used as a wildcard, representing one or more letters. First, two researchers (ZZW and FZG) independently searched the original data on a single day (July 15, 2022) and then discussed the possible differences. Next, the search string was finally determined and confirmed by the two researchers. The final agreement level reached 0.95, showing substantial consistency. The period of interest was 2012–2021. The publication types were confined to original articles and reviews, and only studies published in English were included (18). The screening process is illustrated in Figure 1.

### Data analysis and visualization

The CiteSpace software (5.8. R3), developed by Chen Chaomei from Drexel University (18), was used to visualize collaboration networks (countries/regions, institutions, and authors), analyze co-citations (authors, journals, and references), create dual-map overlays, and determine reference citation bursts and keyword co-occurrences. The specific parameters were as follows: time slicing (from January 2012 to December 2021; years per slice = 1), text processing (title, abstract, author keywords, and keywords plus), node type (one option chosen at a time from a country, institution, author, co-cited journal, co-cited author, keywords, and co-cited reference), link strength (cosine), link scope (within slices), selection criteria (g-index, *k* = 25), and pruning (none).

The journal citation reports (JCR), 2021 impact factor (IF), and JCR division of analyzed journals were obtained from the Web of Science.

## Results

### Analysis of publications and citations

**Figure 2:**
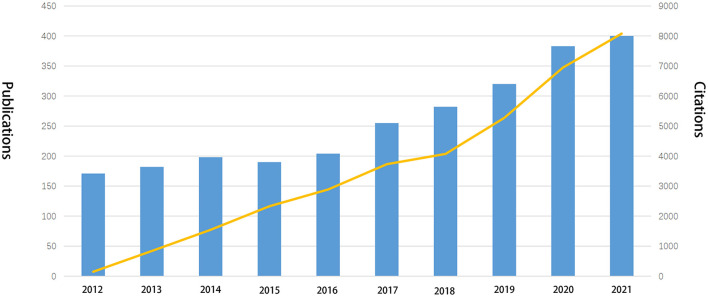
Temporal distribution map of publications and citations.

In total, 2,585 papers related to the management of major trauma were screened for subsequent visualization and analysis. There was generally a growing trend in the numbers and citations of publications from 2012 to 2021, with the lowest in 2012 (*n* = 171, citations = 148) and the highest in 2021 (*n* = 400, citations = 8,079) (Figure 2). Consequently, it is indicated that major trauma is gaining continuous attention and more research is being conducted in this field.

### Distribution map of countries/regions and institutions

**Figure 3:**
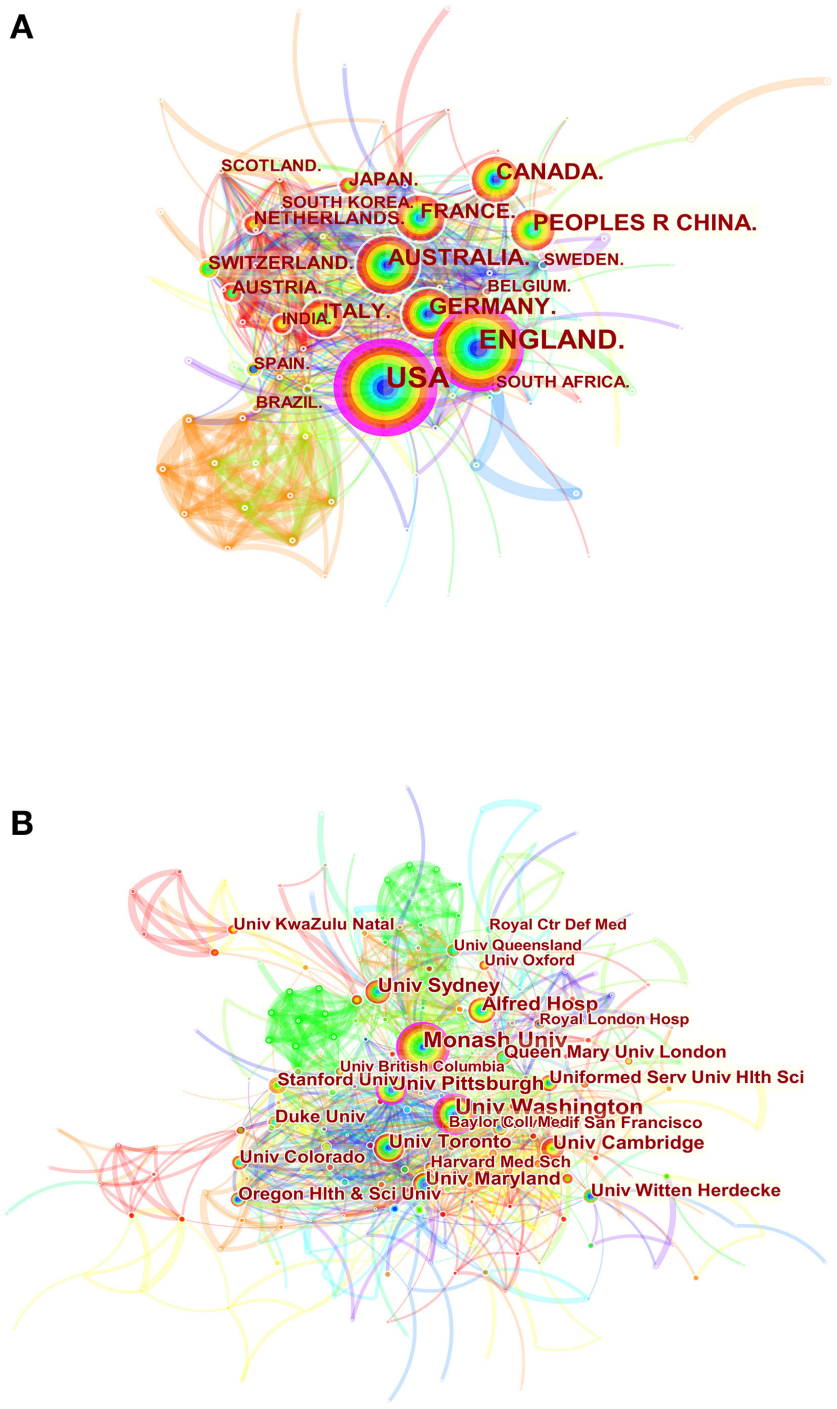
Spatial distribution map of countries/regions **(A)** and institutions **(B)**. Each circle in the diagram represents a nation/institution, with the size of the circle indicating the country/institution's publishing output. The lines that connect the circles represent international collaboration, and the broader the lines, the stronger the cooperation. The colors of the node and line represent different years, and the warmer the color, the more recent the time of publication.

A total of 403 institutions from 110 countries/regions contributed to the research on the management of major trauma. As shown in Table 1, the most productive countries were the USA (753), followed by England (446) and Australia (225). The top three institutions were Monash University (90), the University of Washington (77), and Alfred Hospital (52). In Figure 3, the purple ring indicates the centrality of literature (19). Some countries and institutions had high centralities, such as the USA (0.3), England (0.11), the University of Pittsburgh (0.15), the University of Washington (0.14), and Monash University (0.1). Links between nodes signify relationships of collaboration (19), and dense connections indicate active cooperation among countries and affiliations.

**Table 1:** The top 10 productive countries/regions and institutions.

**Rank**	**Countries/regions**	**Count**	**Centrality**	**Year**	**Rank**	**Institutions**	**City**	**Count**	**Centrality**	**Year**
1	USA	753	0.3	2012	1	Monash Univ	Melbourne	90	0.1	2012
2	England	446	0.11	2012	2	Univ Washington	Seattle	77	0.14	2012
3	Australia	225	0.09	2012	3	Alfred Hosp	Melbourne	52	0.04	2012
4	Germany	183	0.05	2012	4	Univ Pittsburgh	Pittsburgh	48	0.15	2012
5	Peoples R China	155	0.08	2012	5	Univ Sydney	Sydney	43	0.06	2012
6	Canada	150	0.09	2012	6	Univ Toronto	Toronto	42	0.06	2012
7	Italy	135	0.06	2012	7	Univ Cambridge	Cambridge	40	0.06	2016
8	France	135	0.06	2012	8	Univ Maryland	Washington	37	0.05	2012
9	Japan	75	0.04	2013	9	Univ Witten Herdecke	Cologne	32	0.06	2012
10	Netherlands	73	0.03	2012	10	Uniformed Serv Univ Hlth Sci	Bethesda	31	0.04	2013

### Visual analysis of authors and co-cited authors

**Figure 4:**
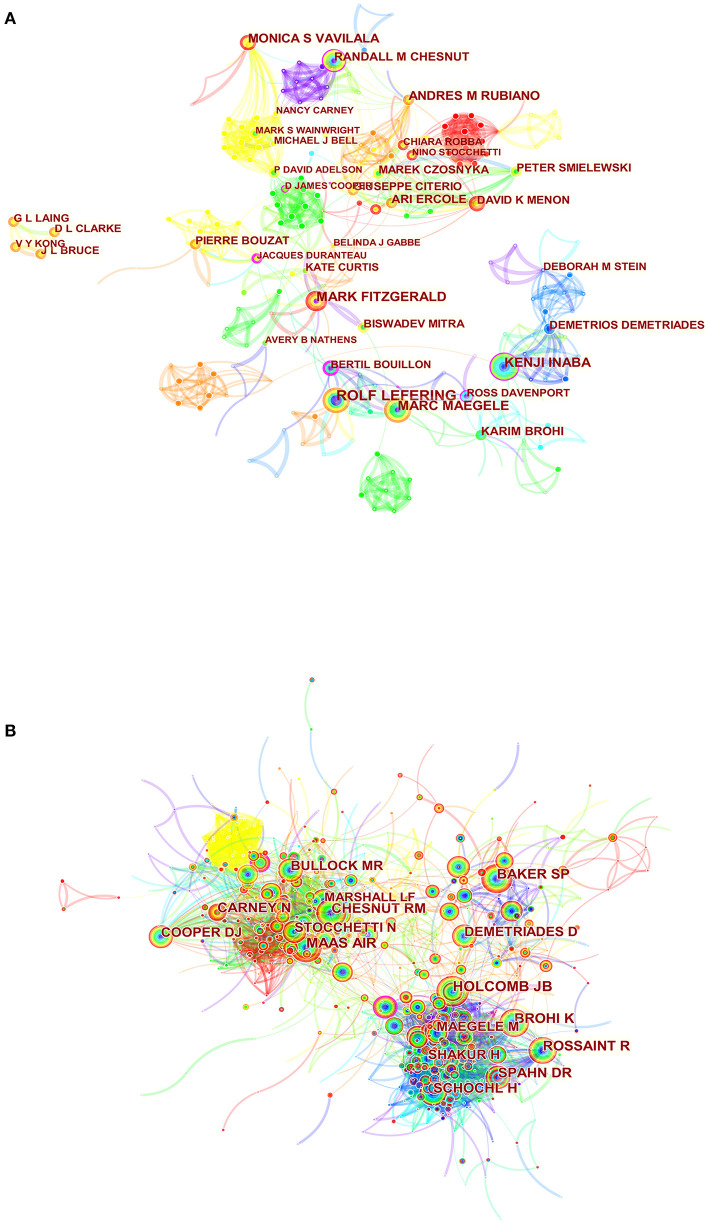
Visual analysis of authors **(A)** and co-cited authors **(B)**. The node size represents the number of studies published by the author, with larger nodes representing a higher number of publications. The closer the collaboration between two writers, the shorter the distance between two nodes. The purple nodes represent early published articles, while the red nodes represent recently published articles.

Each node is labeled by the corresponding author, and the linkage between the two nodes indicates that the two authors cooperated to conduct the research, the details of which were documented in the same paper (20). As shown in Figure 4A and Table 2, 474 authors contributed to the research on the management of major trauma. The most productive author was Rolf Lefering (26), followed by Kenji Inaba (19), Marc Maegele (17), and Mark Fitzgerald (17). The density of the network was 0.0104, indicating that the authors had not formed strong collaborative relations. There are only two authors, for whom the betweenness centrality was more than 0.1: Kenji Inaba (0.15) and Randall M. Chesnut (0.13).

**Table 2:** The top 10 authors and co-citation authors.

**Rank**	**Authors**	**Institutions**	**Count**	**Centrality**	**Year**	**Rank**	**Authors**	**Institutions**	**Citations**	**Centrality**	**Year**
1	Rolf Lefering	University of Witten/Herdecke	26	0.03	2012	1	Holcomb JB	University of Alabama at Birmingham	201	0.02	2012
2	Kenji Inaba	University of Southern California Medical Center	19	0.15	2013	2	Maas Air	Antwerp University Hospital and University of Antwerp	194	0.03	2012
3	Marc Maegele	University of Witten/Herdecke	17	0.03	2012	3	Baker SP	Johns Hopkins University Bloomberg School of Public Health	178	0.01	2012
4	Mark Fitzgerald	The Alfred Hospital	17	0.03	2012	4	Chesnut RM	University of Washington	176	0.03	2012
5	Andres M Rubiano	El Bosque University	16	0.04	2013	5	Schochl H	AUVA Trauma Center Salzburg	170	0.02	2012
6	Monica S Vavilala	University of Washington	15	0.05	2014	6	Spahn DR	University Hospital of Zurich	165	0.01	2012
7	Randall M Chesnut	University of Washington	14	0.13	2012	7	Brohi K	Queen Mary University of London	158	0.01	2012
8	Ari Ercole	Addenbrooke's Hospital	13	0	2016	8	Carney N	Oregon Health and Science University	158	0.01	2017
9	Karim Brohi	The Alfred Hospital	13	0.03	2012	9	Rossaint R	Rhineland-Westfalen Technical University Hospital	153	0.07	2012
10	Demetrios Demetriades	University of Southern California Medical Center	12	0.02	2013	10	Cooper DJ	Monash University	127	0.05	2012

When two scholars are cited in the same publication, an author co-citation relationship occurs. The closer the linkage between the two nodes, the more frequently the two authors are cited in the same paper (20). As shown in Figure 4B and Table 2, the top three most highly cited authors were Holcomb JB (201 citations), Maas Air (194 citations), and Baker SP (178 citations). However, betweenness centralities were relatively low among them (<0.1).

### Visual analysis of journals and co-cited journals

In this study, 2,585 papers concerning the management of major trauma were published in 200 journals, the top 10 of which are listed in Table 3. The most productive journal was *Injury* that published 134 related papers, followed by the *Journal of Trauma and Acute Care Surgery* (92), and the *European Journal of Trauma and Emergency* (61). The journals with the most citations included the *Journal of Trauma* (1,546 citations), *Injury* (1,028 citations), the *Journal of Trauma and Acute Care Surgery* (1,546 citations), *The Lancet* (1,546 citations), and the *New England Journal of Medicine* (1,546 citations). All journals were categorized as Q1 or Q2 in the JCR 2021, except for *Injury*.

**Table 3:** The top 10 journals and co-cited journals.

**Rank**	**Journals**	**Count**	**JCR**	**IF**	**Co-cited journals**	**Co-citations**	**JCR**	**IF**
1	Injury	134	Q3	2.687	The Journal of trauma*	1,546	-	-
2	Journal of trauma and acute care surgery	92	Q2	3.697	Injury	1,028	Q3	2.687
3	European journal of trauma and emergency	61	Q3	2.374	Journal of trauma and acute care surgery	800	Q2	3.697
4	Scandinavian journal of trauma resuscitation	52	Q2	3.803	Lancet	734	Q1	202.731
5	Journal of neurotrauma	45	Q2	4.869	The New England journal of medicine	723	Q1	176.079
6	World neurosurgery	42	Q4	2.21	Critical care medicine	713	Q1	9.296
7	Emergency medicine journal	35	Q3	3.814	Critical care	685	Q1	19.334
8	Critical care	31	Q1	19.334	Journal of neurotrauma	630	Q2	4.869
9	PLOS one	30	Q3	3.752	Annals of surgery	602	Q1	13.787
10	BMJ open	28	Q4	3.006	Journal of neurosurgery	555	Q1	5.408

* This journal was continued by Journal of trauma and acute care surgery since 2011.

**Figure 5:**
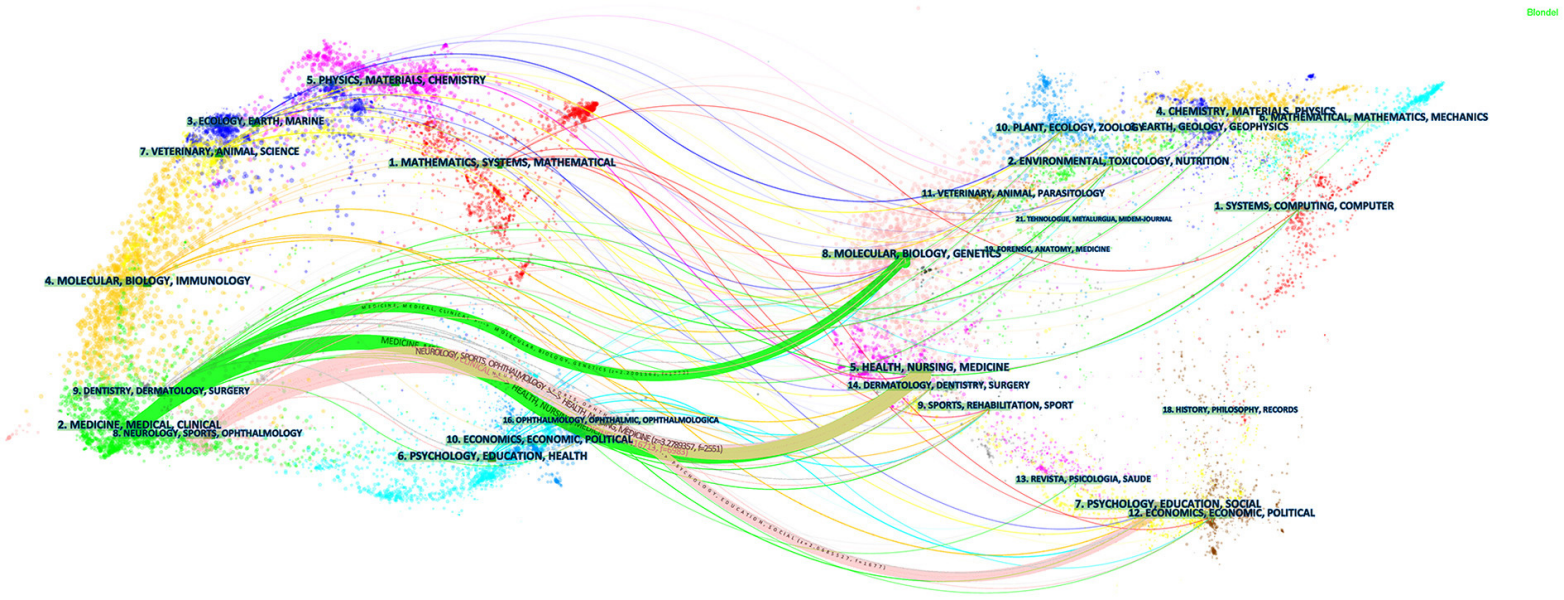
A dual-map overlay of the science mapping literature. The citing journals are on the left, the cited journals are on the right, and the colored path represents the citation relationship. Citation trajectories are distinguished by citing regions' colors. The thickness of these trajectories is proportional to the z-score-scaled frequency of citations.

The dual-map overlay of the literature is shown in Figure 5. In the visual representation, the left clusters represent where the retrieved records are published, whereas the right clusters indicate where they are cited (21). As shown in the figure, our dataset contained four main citation paths. The domains most frequently covering the records were: (1) 2. medicine, medical, clinical and (2) 8. neurology, sports, ophthalmology. The literature was mostly influenced by the following domains: (1) 8. molecular, biology, agents; (2) 5. health, nursing, medicine; and (3) 7. psychology, education, social. Publications from multiple domains contribute to the citation landscapes, indicating a multidisciplinary aspect of opinion mining (21).

### Analysis of co-citation and clustering network

**Figure 6:**
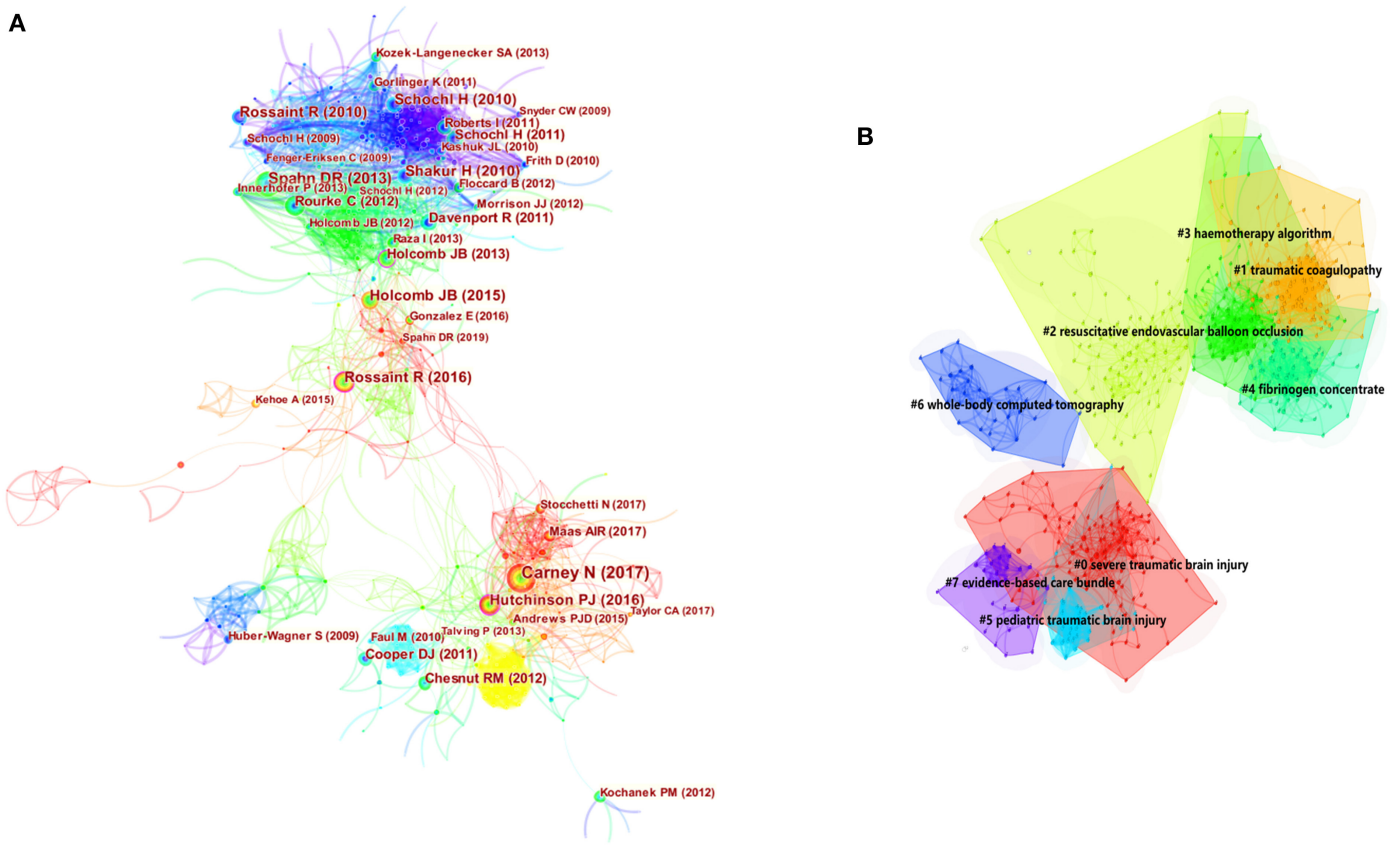
Visual analysis of co-citation **(A)** and clustering network **(B)**. Each circle represents a reference. The size of the circle is proportional to the citation frequency. The link between the two circles represents two references cited in the same article among the citing articles. Line thickness is positively correlated with co-citation frequency.

The generation of reference co-citation map resulted in 761 nodes and 3,382 links (Figure 6A). The first article was published in 2017 by Nancy Carney in terms of citation frequency (22). This article synthesized the available evidence and provided recommendations for the management of severe traumatic brain injury. Another guideline published by Donat R. Spahn in 2013 ranked second (23). The retrospective analysis published by Herbert Schöchl in 2010 (24) ranked third; it pointed out that ROTEM^®^-guided hemostatic therapy, with fibrinogen concentrate as first-line hemostatic therapy and additional prothrombin complex concentrate, was goal-directed and fast. More details pertaining to the top 10 cited references are presented in Table 4.

**Table 4:** Top 10 co-cited references.

**Rank**	**Year**	**Title**	**Journal**	**Co-citations**
1	2017	Guidelines for the Management of Severe Traumatic Brain Injury, Fourth Edition	Neurosurgery	146
2	2013	Management of bleeding and coagulopathy following major trauma: an updated European guideline	Critical care	104
3	2010	Goal-directed coagulation management of major trauma patients using thromboelastometry (ROTEM)-guided administration of fibrinogen concentrate and prothrombin complex concentrate	Critical care	72
4	2015	Transfusion of plasma, platelets, and red blood cells in a 1:1:1 vs. a 1:1:2 ratio and mortality in patients with severe trauma: the PROPPR randomized clinical trial	JAMA	65
5	2010	Management of bleeding following major trauma: an updated European guideline	Critical care	65
6	2016	The European guideline on management of major bleeding and coagulopathy following trauma: fourth edition	Critical care	61
7	2016	Trial of Decompressive Craniectomy for Traumatic Intracranial Hypertension	The New England journal of medicine	61
8	2010	Effects of tranexamic acid on death, vascular occlusive events, and blood transfusion in trauma patients with significant hemorrhage (CRASH-2): a randomized, placebo-controlled trial	Lancet	61
9	2012	A trial of intracranial-pressure monitoring in traumatic brain injury	The New England journal of medicine	55
10	2012	Fibrinogen levels during trauma hemorrhage, response to replacement therapy, and association with patient outcomes	Journal of thrombosis and hemostasis	50

The network has a modularity value of 0.727 and an average silhouette score of 0.9026 that is considered very high, suggesting that the clustering is highly reliable (Figure 6B). The areas of different colors represent the time when the co-citation links appeared for the first time. The brighter the color, the closer the average year of one cluster was to the present (20, 25). Clusters were labeled with title terms extracted from the citing articles, using the log-likelihood ratio (LLR) algorithm. Figure 6B shows eight clusters, including #0 severe traumatic brain injury, #1 traumatic coagulopathy, #2 resuscitative endovascular balloon occlusion, #3 hemotherapy algorithm, #4 fibrinogen concentrate, #5 pediatric traumatic brain injury, #6 whole-body computed tomography, and #7 evidence-based care bundles. The color of the convex hull of each cluster indicates recent research topics, including cluster #0 severe traumatic brain injury, #1 traumatic coagulopathy, and #2 resuscitative endovascular balloon occlusion.

**Figure 7:**
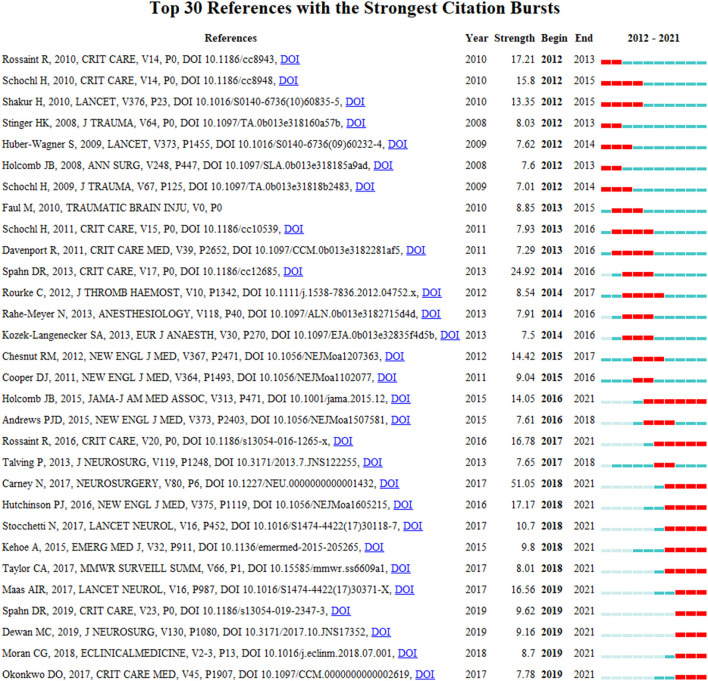
Visual analysis of references bursts. The intensity value reflects the cited frequency. The red bar indicates citation frequency; green bars indicate fewer citations.

The top 30 references with the strongest citation bursts between 2012 and 2021 were identified (Figure 7). References with strong values in the strength column tend to be significant milestones in science mapping research. For instance, in this study, the first milestone paper was a guideline for the management of bleeding and coagulopathy following a major traumatic injury (23), and the next milestone was a guideline for the management of severe traumatic brain injuries (22).

### Visual analysis of keywords

**Figure 8:**
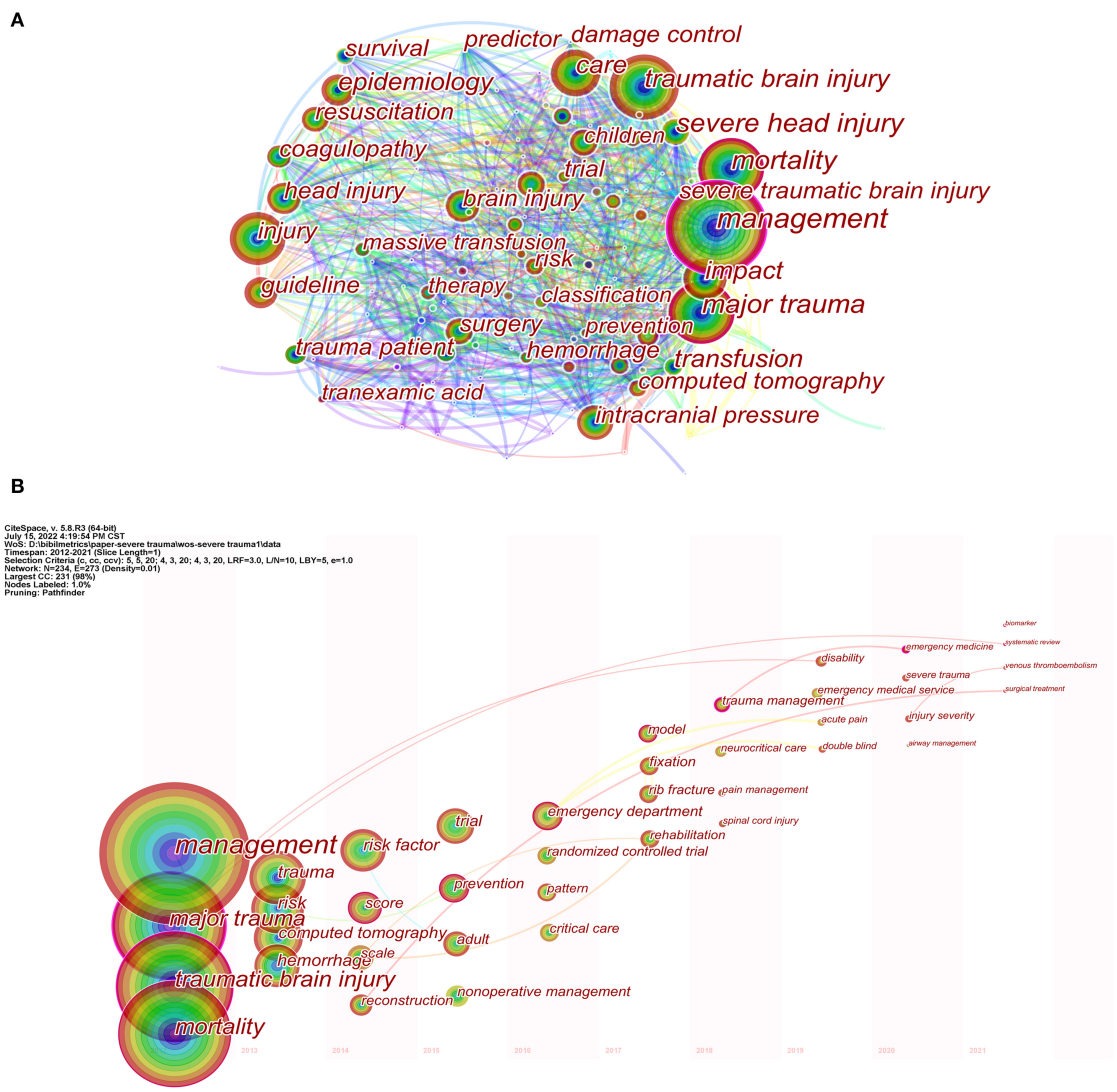
**(A)** Keyword co-occurrence network. Different colors of the circles indicate the average year of the studies according to the bar on the lower right corner. **(B)** Keywords “timezone view” in management of major trauma research.

We generated a network map of keywords consisting of 514 nodes and 4,223 links (Figure 8A). In the top 20 keywords listed in Table 5, the popular keywords were “management,” “traumatic brain injury,” “mortality,” “major trauma,” and “injury,” all of which had high citations.

**Table 5:** The top 20 keywords.

**Rank**	**Keywords**	**Citations**	**Centrality**	**Year**
1	Management	983	0.22	2012
2	Traumatic brain injury	372	0.07	2012
3	Mortality	370	0.12	2012
4	Major trauma	320	0.2	2012
5	Injury	249	0.06	2012
6	Outcome	242	0.11	2012
7	Care	216	0.07	2012
8	Impact	173	0.06	2012
9	Guideline	150	0.05	2012
10	Epidemiology	144	0.06	2012
11	Intracranial pressure	142	0.02	2012
12	Head injury	141	0.05	2012
13	Severe head injury	126	0.08	2012
14	Brain injury	119	0.04	2012
15	Children	119	0.02	2012
16	Surgery	103	0.03	2012
17	Resuscitation	100	0.05	2012
18	Decompressive craniectomy	96	0.01	2012
19	Trauma	87	0.01	2012
20	Risk	86	0.04	2012

The keyword “timezone view” displays the evolution of high-frequency keywords. Figure 8B shows the research hotspots in the management of major trauma. From 2012 to 2016, research keywords focused on “management,” “trauma,” “risk factor,” “trial,” and “emergency department”. These keywords indicate the research mainly involved in clinical practice or trials. From 2017 to 2021, the primary terms were “model,” “trauma management,” “neurocritical care,” “emergency medicine,” and “biomarker.” These results indicate that researchers may pay more attention to advanced technology, newer methods, strict administration, and fundamental research.

**Figure 9:**
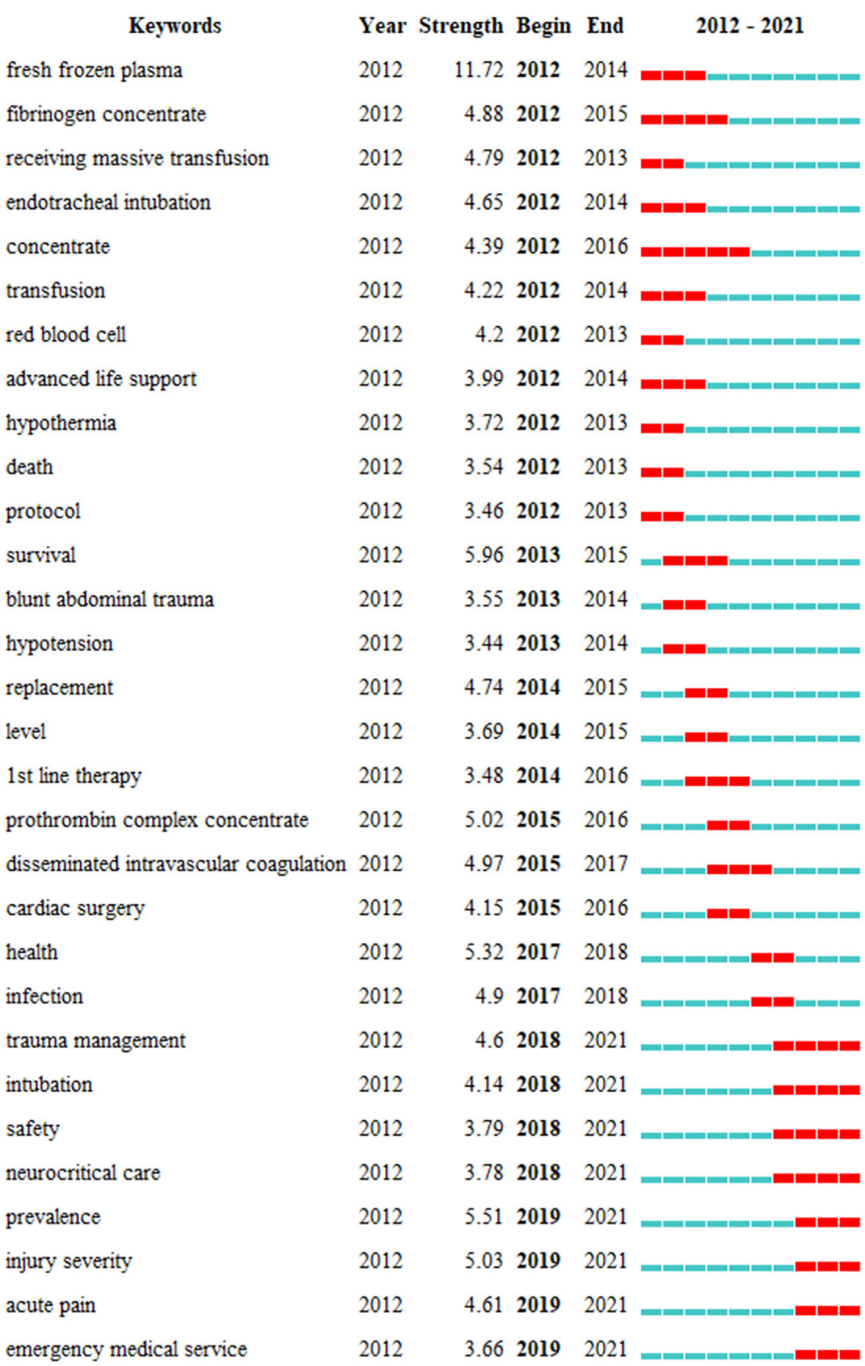
Top 30 keywords with citation burst (sorted by the beginning year of the burst).

The top 30 keywords with the strongest citation bursts are shown in Figure 9. The keyword “fresh frozen plasma,” emerging in 2012, showed the strongest citation burst of 11.72. The most recent burst keywords were “trauma management,” “neurocritical care,” “injury severity,” and “emergency medical service,” revealing research trending over time and reflecting future hotspots (26).

## Discussion

### General information

Major trauma considerably affects health at both the individual and population levels (27). Previous literature reported bibliometric analysis of severe traumatic brain injury (28), spinal cord injury (29), traumatology (30), etc. As bibliometric studies concerning major trauma are scarce, this study, to the best of our knowledge, is the first bibliometric analysis of the dynamic structures and emerging trends in the management of major trauma between 2012 and 2021. After the screening process, we found that over the last decade, a total of 474 authors from 403 institutions in 110 countries published 2,585 papers related to the management of major trauma in 200 academic journals. We used CiteSpace to evaluate the networks of co-authors' countries/institutions, co-authorship, author co-citations, journal co-citations, document co-citations, and co-occurring keywords, to identify the knowledge domain and frontier trends in the management of major trauma.

The analysis of the network of co-authors' countries/regions and institutions (Table 1; Figure 3) showed that the USA, England, and Australia were the top three nations in terms of the number of publications related to the management of major trauma. The USA has the highest betweenness centrality (0.3), indicating that it plays a key role in bridging national cooperation networks worldwide. Meanwhile, only two Asian countries—China and Japan—were ranked among the top 10 productive countries, indicating that Asian countries need further investment in the field of research on severe trauma. Monash University published the highest number of papers. We also found extensive connections between institutions, indicating significant collaborative contributions to this research field.

In the analysis of authors and co-cited authors, Rolf Lefering, a researcher from the University of Witten/Herdecke, made the most contributions with 26 published studies, followed by Kenji Inaba from the University of Southern California Medical Center, with 19 articles. These two authors have been extensively involved in the clinical research of severe trauma, such as whole-body CT in polytrauma (31), the administration of tranexamic acid and fibrinogen concentrate in patients with trauma (32, 33), intracranial pressure monitoring in severe head injury (34), massive transfusion protocol (35), and emergency operation (36). Holcomb JB from the University of Alabama at Birmingham received the most co-citations (201 citations) and is active in the field of severe trauma research (37–39).

As shown in Table 3, *Injury* published the highest number of papers, followed by the *Journal of Trauma and Acute Care Surgery* and the *European Journal of Trauma and Emergency*. Papers published in high-IF journals, such as *The Lancet*, the *New England Journal of Medicine*, and *Critical Care*, had more co-citations and the findings of this study provide a theoretical basis for future research (26). As shown in Figure 5, there are four main citation paths in our dataset, indicating a multidisciplinary aspect of this field, as publications in multiple domains have contributed to the citation landscape.

As shown in Table 4, the top 10 co-cited references mainly focused on the management of traumatic brain injury (22, 40) and trauma hemorrhage (4, 23, 24, 41). To automatically label the clusters of cited references, we extracted candidate terms from the titles and abstracts of the citing articles. The labels extracted by the LLR tended to reflect a unique aspect of the cluster. The purple and blue nodes represent early clustering labels that included pediatric traumatic brain injury (#5), whole-body computed tomography (#6), and evidence-based care bundle (#7), whereas the red and yellow nodes represent recent clustering labels, such as severe traumatic brain injury (#0), traumatic coagulopathy (#1), and resuscitative endovascular balloon occlusion (#2).

### Research hotspots and emerging topics

Reference clusters and citation bursts can characterize the emerging topics in the discipline. The two main themes indicate the current hot topics in major trauma research.

In cluster #0, the literature largely reported on the management of severe traumatic brain injury (42–47). The management of traumatic brain injury (TBI) has changed over the past decade; a multimodal approach is now being applied in detecting and treating the pathophysiological derangements. The theoretical highlights include initial pre- and in-hospital resuscitation, secondary injury management (management of elevated intracranial pressure, management of cerebral perfusion pressure, and multimodality monitoring), and extracranial complications (respiratory management, fluid management, nutrition management, mobilization and rehabilitation, etc.) (48, 49). The increasing availability of big data and computational science pave the way toward more accurate neuroprognostication (50). Experimental efforts to promote repair in TBI have been made including cell-based or gene therapies (51), acellular scaffolds (52), endogenous growth-related factors (48), etc.

In clusters #1 and #2, the literature focused on management of massive hemorrhage, specifically traumatic coagulopathy and resuscitative endovascular balloon occlusion. Management of massive hemorrhage over the past decade has evolved to now deliver a package of hemostatic resuscitation including surgical or radiological control of bleeding; regular monitoring of hemostasis; advanced critical care support; and avoidance of the lethal triad of hypothermia, academia, and coagulopathy (53). Resuscitative endovascular balloon occlusion of the aorta (REBOA) is growingly utilized in trauma resuscitation for patients with life-threatening hemorrhage below the diaphragm (54), and is also available in a few pre-hospital critical care teams (55). Traumatic coagulopathy describes abnormal coagulation processes that are attributable to trauma. In the early hours of traumatic coagulopathy development, hypocoagulability is typically present, resulting in bleeding, whereas later traumatic coagulopathy is characterized by a hypercoagulable state associated with venous thromboembolism and multiple organ failure (56).

Keyword analysis helps identify research hotspots and predicts developing trends in the field (57). As indicated in Figures 8B, 9, the following keywords may indicate the recent focus and research hotspots: “trauma management,” “neurocritical care,” “injury severity,” and “emergency medical service.”

Neurocritical care forms an essential component of trauma management and an emerging field within critical care medicine. Intracranial pressure monitoring is now frequently discussed in the clinical care of many life-threatening brain insults; however, related technologies and management remain a high priority in neurosurgery and neurocritical care (58). Electroencephalography (EEG) is an extremely sophisticated brain monitoring tool that is extensively employed in neurocritical care; the emerging applications of EEG include seizure detection, ischemia monitoring, detection of cortical spreading depolarizations, assessment of consciousness and prognostication (59). Brain injury in children is a major public health problem; pediatric neurocritical care involves assessment, monitoring, and protection of the brain (60). More practice guidelines and establishment of multidisciplinary services are needed for improving healthcare for brain injuries (61).

The severity of injury is assessed by ISS that is associated with methods and description of studies concerning major trauma. In recent years, it is universally employed in scientific research related to major trauma. For instance, Versluijs et al. (62) reviewed the association between trauma severity and post-injury symptoms of depression; Santos et al. (63) predicted the severity of crash injury by investigating machine learning algorithms.

Emergency medical service (EMS) and pre-hospital rescue management are now globally confronted with challenges, including rising number of calls, overcrowding in emergency departments, difficulty in human resource management, etc. (64, 65). However, new EMS resources such as community paramedics and telemedical support systems offer opportunities to strengthen competencies in patient care (66). Consequently, increasing academization and research in this field are welcomed.

### Limitations

This study had the following limitations. According to a study, it is acknowledged that WoSCC is the recommended database for bibliometric analysis (18). Consequently, data were collected from the WoSCC database, whereas data from other sources such as PubMed, Google Scholar, and Embase were not included. As several newly published and potentially high-impact studies may not have been included in our study, the emerging hotspots and trends in major trauma research may vary with bibliometric data updates.

## Conclusion

In conclusion, this bibliometric study provides a comprehensive analysis of dynamic structures and emerging trends in major trauma research using the visualization software, CiteSpace. Based on our findings, the leading countries are the United States, England, and Australia, while Asian countries need more investment in the research field. Management of severe traumatic brain injury and massive hemorrhage, neurocritical care, injury severity, and emergency medical service are emerging and promising research hotspots. Though current information is crucial for future research and policy making in this area, more evidence-based guidelines are needed for clinical practice in the management of major trauma.

## Data availability statement

The raw data supporting the conclusions of this article will be made available by the authors, without undue reservation.

## Author contributions

ZD and TW: design of this study and supervision. ZW: literature search and data analysis. FG, ZD, and TW: manuscript writing and editing. All authors approved the final version of the article.

## Funding

This study was supported by Peking University People's Hospital Scientific Research Development Funds (Grant/Award Number: RDJP2022-06).

## Conflict of interest

The authors declare that the research was conducted in the absence of any commercial or financial relationships that could be construed as a potential conflict of interest.

## Publisher's note

All claims expressed in this article are solely those of the authors and do not necessarily represent those of their affiliated organizations, or those of the publisher, the editors and the reviewers. Any product that may be evaluated in this article, or claim that may be made by its manufacturer, is not guaranteed or endorsed by the publisher.

## References

[1] Li YH Yeung JHH Hung KKC Lai CY Leung LY Cheng CH. Impact of AIS 2015 versus 1998 on injury severity scoring and mortality prediction - single centre retrospective comparison study. Am J Emerg Med. (2022) 60:73–7. 10.1016/j.ajem.2022.07.050 35908299

[2] Costa A Carron PN Zingg T Roberts I Ageron FX Swiss Trauma R. Early identification of bleeding in trauma patients: external validation of traumatic bleeding scores in the Swiss Trauma Registry. Critical care. (2022) 26:296. 10.1186/s13054-022-04178-8 36171598 PMC9520811

[3] Stiell IG Nesbitt LP Pickett W Munkley D Spaite DW Banek J. The OPALS Major Trauma Study: impact of advanced life-support on survival and morbidity. CMAJ. (2008) 178:1141–52. 10.1503/cmaj.071154 18427089 PMC2292763

[4] Rossaint R Bouillon B Cerny V Coats TJ Duranteau J Fernandez-Mondejar E. The European guideline on management of major bleeding and coagulopathy following trauma: fourth edition. Critical care. (2016) 20:100. 10.1186/s13054-016-1265-x 27072503 PMC4828865

[5] Martino C Russo E Santonastaso DP Gamberini E Bertoni S Padovani E. Long-term outcomes in major trauma patients and correlations with the acute phase. World J Emerg Surg. (2020) 15:6. 10.1186/s13017-020-0289-3 31956336 PMC6958936

[6] McCullough AL Haycock JC Forward DP Moran CG. Early management of the severely injured major trauma patient. Br J Anaesth. (2014) 113:234–41. 10.1093/bja/aeu235 25038155

[7] Tisherman SA Stein DM. ICU management of trauma patients. Crit Care Med. (2018) 46:1991–7. 10.1097/CCM.0000000000003407 30199391

[8] O'Reilly GM Curtis K Kim Y Mitra B Hunter K Ryder C. The Australian traumatic brain injury national data (ATBIND) project: a mixed methods study protocol. Med J Aust. (2022) 217:361–5. 10.5694/mja2.51674 35922394 PMC9805146

[9] Xu S Shi B Yuxian J He M Yang P Xu W. Comparative analysis of the wounded in patients and deaths in a hospital following the three major earthquakes in Western China. Front Public Health. (2022) 10:775130. 10.3389/fpubh.2022.775130 35875049 PMC9304578

[10] Liu GY Haudenschild DR Lewis JS. Intra-articular injection of flavopiridol-loaded microparticles for treatment of post-traumatic osteoarthritis. Acta Biomaterialia. (2022) 149:347–58. 10.1016/j.actbio.2022.06.042 35779774 PMC10281459

[11] Luo H Cai Z Huang Y Song J Ma Q Yang X. Study on pain catastrophizing from 2010 to 2020: a bibliometric analysis via CiteSpace. Front Psychol. (2021) 12:759347. 10.3389/fpsyg.2021.759347 34975649 PMC8718514

[12] Kokol P Blazun Vosner H Zavrsnik J. Application of bibliometrics in medicine: a historical bibliometrics analysis. Health Info Libr J. (2021) 38:125–38. 10.1111/hir.12295 31995273

[13] Nonboe MH Lynge E. How can we use bibliometric analysis to guide research forward?-an editorial for “Research trends and hotspots on human papillomavirus: a bibliometric analysis of 100 most-cited articles”. Ann Transl Med. (2022) 10:849. 10.21037/atm-2022-30 36111040 PMC9469111

[14] Zhou Q Kong HB He BM Zhou SY. Bibliometric analysis of bronchopulmonary dysplasia in extremely premature infants in the web of science database using CiteSpace software. Front Pediatr. (2021) 9:705033. 10.3389/fped.2021.705033 34490163 PMC8417835

[15] Qiao G Cao Y Chen Q Jia Q. Understanding family tourism: a perspective of bibliometric review. Front Psychol. (2022) 13:937312. 10.3389/fpsyg.2022.937312 35859832 PMC9291435

[16] Yang W Wang S Chen C Leung HH Zeng Q Su X. Knowledge mapping of enterprise network research in China: a visual analysis using CiteSpace. Front Psychol. (2022) 13:898538. 10.3389/fpsyg.2022.898538 35846692 PMC9282046

[17] Jiang B Feng C Hu H George D Huang T Li Z. Traditional Chinese exercise for neurodegenerative diseases: a bibliometric and visualized analysis with future directions. Front Aging Neurosci. (2022) 14:932924. 10.3389/fnagi.2022.932924 35832067 PMC9271864

[18] Cheng K Guo Q Shen Z Yang W Wang Y Sun Z. Bibliometric analysis of global research on cancer photodynamic therapy: focus on nano-related research. Front Pharmacol. (2022) 13:927219. 10.3389/fphar.2022.927219 35784740 PMC9243586

[19] Zheng J Hou M Liu L Wang X. Knowledge structure and emerging trends of telerehabilitation in recent 20 years: a bibliometric analysis via CiteSpace. Front Public Health. (2022) 10:904855. 10.3389/fpubh.2022.904855 35795695 PMC9251196

[20] Su ZW Zhang MY Wu WB. Visualizing sustainable supply chain management: a systematic scientometric review. Sustainability. (2021) 13:4409. 10.3390/su13084409

[21] Zhu YJ Kim MC Chen CM. An investigation of the intellectual structure of opinion mining research. Inform Res. (2017) 22.

[22] Carney N Totten AM O'Reilly C Ullman JS Hawryluk GW Bell MJ. Guidelines for the management of severe traumatic brain injury, fourth edition. Neurosurgery. (2017) 80:6–15. 10.1227/NEU.0000000000001432 27654000

[23] Spahn DR Bouillon B Cerny V Coats TJ Duranteau J Fernandez-Mondejar E. Management of bleeding and coagulopathy following major trauma: an updated European guideline. Crit Care. (2013) 17:R76. 10.1186/cc12685 23601765 PMC4056078

[24] Schöchl H Nienaber U Hofer G Voelckel W Jambor C Scharbert G. Goal-directed coagulation management of major trauma patients using thromboelastometry (ROTEM)-guided administration of fibrinogen concentrate and prothrombin complex concentrate. Crit Care. (2010) 14:R55. 10.1186/cc8948 20374650 PMC2887173

[25] Chen C. Science mapping: a systematic review of the literature. J Data Inform Sci. (2017) 2:1–40. 10.1515/jdis-2017-0006

[26] Song L Zhang J Ma D Fan Y Lai R Tian W. A bibliometric and knowledge-map analysis of macrophage polarization in atherosclerosis from 2001 to 2021. Front Immunol. (2022) 13:910444. 10.3389/fimmu.2022.910444 35795675 PMC9250973

[27] Holtslag HR van Beeck EF Lichtveld RA Leenen LP Lindeman E van der Werken C. Individual and population burdens of major trauma in the Netherlands. Bull World Health Organ. (2008) 86:111–7. 10.2471/BLT.06.033803 18297165 PMC2647381

[28] Li L Ma X Pandey S Deng X Chen S Cui D. The most-cited works in severe traumatic brain injury: a bibliometric analysis of the 100 most-cited articles. World Neurosurg. (2018) 113:e82–7. 10.1016/j.wneu.2018.01.164 29409928

[29] Kiraz M Demir E. A bibliometric analysis of publications on spinal cord injury during 1980-2018. World Neurosurg. (2020) 136:e504–13. 10.1016/j.wneu.2020.01.064 31954906

[30] Dokur M Uysal E. Top 100 cited articles in traumatology: a bibliometric analysis. Ulus Travma Acil Cerrahi Derg. (2018) 24:294–302. 10.5505/tjtes.2017.74857 30028485

[31] Huber-Wagner S Biberthaler P Haberle S Wierer M Dobritz M Rummeny E. Whole-body CT in haemodynamically unstable severely injured patients–a retrospective, multicentre study. PLoS ONE. (2013) 8:e68880. 10.1371/journal.pone.0068880 23894365 PMC3722202

[32] Wafaisade A Lefering R Bouillon B Bohmer AB Gassler M Ruppert M. Prehospital administration of tranexamic acid in trauma patients. Crit Care. (2016) 20:143. 10.1186/s13054-016-1322-5 27176727 PMC4866028

[33] Wafaisade A Lefering R Maegele M Brockamp T Mutschler M Lendemans S. Administration of fibrinogen concentrate in exsanguinating trauma patients is associated with improved survival at 6 hours but not at discharge. J Trauma Acute Care Surg. (2013) 74:387–3. 10.1097/TA.0b013e31827e2410 23354229

[34] Talving P Karamanos E Teixeira PG Skiada D Lam L Belzberg H. Intracranial pressure monitoring in severe head injury: compliance with Brain Trauma Foundation guidelines and effect on outcomes: a prospective study. J Neurosurg. (2013) 119:1248–54. 10.3171/2013.7.JNS122255 23971954

[35] Nosanov L Inaba K Okoye O Resnick S Upperman J Shulman I. The impact of blood product ratios in massively transfused pediatric trauma patients. Am J Surg. (2013) 206:655–60. 10.1016/j.amjsurg.2013.07.009 24011571

[36] Matsushima K Inaba K Siboni S Skiada D Strumwasser AM Magee GA. Emergent operation for isolated severe traumatic brain injury: does time matter? J Trauma Acute Care Surg. (2015) 79:838–42. 10.1097/TA.0000000000000719 26317818

[37] Hashmi ZG Jansen JO Kerby JD Holcomb JB. Nationwide estimates of the need for prehospital blood products after injury. Transfusion. (2022) 62(Suppl. 1):S203–10. 10.1111/trf.16991 35753065

[38] Yazer MH Cap AP Glassberg E Green L Holcomb JB Khan MA. Toward a more complete understanding of who will benefit from prehospital transfusion. Transfusion. (2022) 62:1671–9. 10.1111/trf.17012 35796302

[39] Gelbard RB Griffin RL Reynolds L Abraham P Warner J Hu P. Over-transfusion with blood for suspected hemorrhagic shock is not associated with worse clinical outcomes. Transfusion. (2022) 62(Suppl. 1):S177–84. 10.1111/trf.16978 35753037

[40] Hutchinson PJ Kolias AG Timofeev IS Corteen EA Czosnyka M Timothy J. Trial of decompressive craniectomy for traumatic intracranial hypertension. N Engl J Med. (2016) 375:1119–30. 10.1056/NEJMoa1605215 27602507

[41] Holcomb JB Tilley BC Baraniuk S Fox EE Wade CE Podbielski JM. Transfusion of plasma, platelets, and red blood cells in a 1:1:1 vs. a 1:1:2 ratio and mortality in patients with severe trauma: the PROPPR randomized clinical trial. JAMA. (2015) 313:471–82. 10.1001/jama.2015.12 25647203 PMC4374744

[42] Zeiler FA Iturria-Medina Y Thelin EP Gomez A Shankar JJ Ko JH. Integrative neuroinformatics for precision prognostication and personalized therapeutics in moderate and severe traumatic brain injury. Front Neurol. (2021) 12:729184. 10.3389/fneur.2021.729184 34557154 PMC8452858

[43] Kochanek PM Tasker RC Carney N Totten AM Adelson PD Selden NR. Guidelines for the management of pediatric severe traumatic brain injury, third edition: update of the brain trauma foundation guidelines. Pediatr Crit Care Med. (2019) 20:S1–S82. 10.1097/PCC.0000000000001735 30829890

[44] Chau CYC Mediratta S McKie MA Gregson B Tulu S Ercole A. Optimal timing of external ventricular drainage after severe traumatic brain injury: a systematic review. J Clin Med. (2020) 9:1996. 10.3390/jcm9061996 32630454 PMC7356750

[45] Ozoner B. Cranioplasty following severe traumatic brain injury: role in neurorecovery. Curr Neurol Neurosci Rep. (2021) 21:62. 10.1007/s11910-021-01147-6 34674047

[46] Batson C Froese L Gomez A Sainbhi AS Stein KY Alizadeh A. Impact of age and biological sex on cerebrovascular reactivity in adult moderate/severe traumatic brain injury: an exploratory analysis. Neurotrauma Rep. (2021) 2:488–501. 10.1089/neur.2021.0039 34901944 PMC8655816

[47] Takahashi CE Virmani D Chung DY Ong C Cervantes-Arslanian AM. Blunt and penetrating severe traumatic brain injury. Neurol Clin. (2021) 39:443–69. 10.1016/j.ncl.2021.02.009 33896528

[48] Meyfroidt G Bouzat P Casaer MP Chesnut R Hamada SR Helbok R. Management of moderate to severe traumatic brain injury: an update for the intensivist. Intensive Care Med. (2022) 48:649–66. 10.1007/s00134-022-06702-4 35595999

[49] Krishnamoorthy V Komisarow JM Laskowitz DT Vavilala MS. Multiorgan dysfunction after severe traumatic brain injury: epidemiology, mechanisms, and clinical management. Chest. (2021) 160:956–64. 10.1016/j.chest.2021.01.016 33460623 PMC8448997

[50] Dijkland SA Foks KA Polinder S Dippel DWJ Maas AIR Lingsma HF. Prognosis in moderate and severe traumatic brain injury: a systematic review of contemporary models and validation studies. J Neurotrauma. (2020) 37:1–13. 10.1089/neu.2019.6401 31099301

[51] Burns TC Quinones-Hinojosa A. Regenerative medicine for neurological diseases-will regenerative neurosurgery deliver? BMJ. (2021) 373:n955. 10.1136/bmj.n955 34162530

[52] Latchoumane CV Betancur MI Simchick GA Sun MK Forghani R Lenear CE. Engineered glycomaterial implants orchestrate large-scale functional repair of brain tissue chronically after severe traumatic brain injury. Sci Adv. (2021) 7:eabe0207. 10.1126/sciadv.abe0207 33674306 PMC7935369

[53] Shah A Kerner V Stanworth SJ Agarwal S. Major haemorrhage: past, present and future. Anaesthesia. (2022). 10.1111/anae.15866. [Epub ahead of print]. 36089857 PMC10087440

[54] Aoki M Abe T. Traumatic cardiac arrest: scoping review of utilization of resuscitative endovascular balloon occlusion of the aorta. Front Med. (2022) 9:888225. 10.3389/fmed.2022.888225 35783650 PMC9243328

[55] Ter Avest E Carenzo L Lendrum RA Christian MD Lyon RM Coniglio C. Advanced interventions in the pre-hospital resuscitation of patients with non-compressible haemorrhage after penetrating injuries. Crit Care. (2022) 26:184. 10.1186/s13054-022-04052-7 35725641 PMC9210796

[56] Moore EE Moore HB Kornblith LZ Neal MD Hoffman M Mutch NJ. Trauma-induced coagulopathy. Nat Rev Dis Primers. (2021) 7:30. 10.1038/s41572-021-00264-3 33927200 PMC9107773

[57] Li X Wei W Wang Y Wang Q Liu Z. Global trend in the research and development of acupuncture treatment on Parkinson's disease from 2000 to 2021: a bibliometric analysis. Front Neurol. (2022) 13:906317. 10.3389/fneur.2022.906317 35873762 PMC9305197

[58] Hawryluk GWJ Citerio G Hutchinson P Kolias A Meyfroidt G Robba C. Intracranial pressure: current perspectives on physiology and monitoring. Intensive Care Med. (2022) 48:1471–81. 10.1007/s00134-022-06786-y 35816237

[59] Alkhachroum A Appavu B Egawa S Foreman B Gaspard N Gilmore EJ. Electroencephalogram in the intensive care unit: a focused look at acute brain injury. Intensive Care Med. (2022) 48:1443–62. 10.1007/s00134-022-06854-3 35997792 PMC10008537

[60] Brown KL Agrawal S Kirschen MP Traube C Topjian A Pressler R. The brain in pediatric critical care: unique aspects of assessment, monitoring, investigations, and follow-up. Intensive Care Med. (2022) 48:535–47. 10.1007/s00134-022-06683-4 35445823 PMC10082392

[61] Sarnaik AA. Pediatric neurocritical care. Pediatr Clin North Am. (2022) 69:415–24. 10.1016/j.pcl.2022.01.007 35667754

[62] Versluijs Y van Ravens TW Krijnen P Ring D Schipper IB. Systematic review of the association between trauma severity and postinjury symptoms of depression. World J Surg. (2022). 10.1007/s00268-022-06750-3 [Epub ahead of print]. 36175650 PMC9636287

[63] Santos K Dias JP Amado C. A literature review of machine learning algorithms for crash injury severity prediction. J Safety Res. (2022) 80:254–69. 10.1016/j.jsr.2021.12.007 35249605

[64] Sartini M Carbone A Demartini A Giribone L Oliva M Spagnolo AM e al. Overcrowding in emergency department: causes, consequences, and solutions-a narrative review. Healthcare. (2022) 10:1625. 10.3390/healthcare10091625 36141237 PMC9498666

[65] Lauer D Bandlow S Rathje M Seidl A Karutz H. Changes and developments in emergency medical services: key challenges for rescue management. Bundesgesundheitsblatt Gesundheitsforschung Gesundheitsschutz. (2022) 65:987–95. 10.1007/s00103-022-03588-x 36112196 PMC9483533

[66] Dahlmann P Böbel S Frieß C Neuerer M. Educational perspectives in emergency paramedicine: interdisciplinary discourse on education, professional practice, and challenges in the field of emergency medical services. Bundesgesundheitsblatt Gesundheitsforschung Gesundheitsschutz. (2022) 65:1059–66. 10.1007/s00103-022-03574-3 35982327

